# Microbial inoculants for soil restoration: a risk-proportional stewardship framework integrating strain-resolved genomics and adaptive governance

**DOI:** 10.1093/sumbio/qvag036

**Published:** 2026-08-17

**Authors:** Anna Edlund, Gwyn A Beattie, Joana Falcao Salles, Jack A Gilbert, Janet K Jansson, Jay T Lennon, Jennifer B H Martiny, Ian R Sanders, Christopher W Schadt, Carsten Suhr Jacobsen, Matthew B Sullivan

**Affiliations:** The Soil Stars, Applied Microbiology International, Cambridge CB12LA, United Kingdom; Oath Inc., San Francisco, CA 94129, United States; The Soil Stars, Applied Microbiology International, Cambridge CB12LA, United Kingdom; Department of Plant Pathology, Entomology and Microbiology, Iowa State University, Ames, IA 50011, United States; The Soil Stars, Applied Microbiology International, Cambridge CB12LA, United Kingdom; Microbial Ecology Cluster, Genomics Research in Ecology and Evolution in Nature (GREEN), Groningen Institute for Evolutionary Life Sciences (GELIFES), University of Groningen, Groningen 9747AG, The Netherlands; The Netherlands & Department of Microbial Ecology, Centre for Life Sciences, University of Groningen, 9700 Groningen, The Netherlands; The Soil Stars, Applied Microbiology International, Cambridge CB12LA, United Kingdom; Oath Inc., San Francisco, CA 94129, United States; Department of Pediatrics, University of California San Diego, La Jolla, CA 92037, United States; The UC San Diego Soil Health Center, Scripps Institution of Oceanography, University of California San Diego, La Jolla, CA 92037, United States; The Soil Stars, Applied Microbiology International, Cambridge CB12LA, United Kingdom; Oath Inc., San Francisco, CA 94129, United States; The Soil Stars, Applied Microbiology International, Cambridge CB12LA, United Kingdom; Department of Biology, Indiana University, Bloomington, IN 47405, United States; The Soil Stars, Applied Microbiology International, Cambridge CB12LA, United Kingdom; Department of Ecology & Evolutionary Biology, University of California, Irvine, CA 92697, United States; Department of Biotechnology and Biomedicine, Technical University of Denmark, Kongens Lyngby DK-2800, Denmark; The Soil Stars, Applied Microbiology International, Cambridge CB12LA, United Kingdom; Department of Ecology and Evolution, University of Lausanne, Lausanne CH-1015, Switzerland; The Soil Stars, Applied Microbiology International, Cambridge CB12LA, United Kingdom; Biosciences Division, Oak Ridge National Laboratory, Oak Ridge, TN 37831, United States; The Soil Stars, Applied Microbiology International, Cambridge CB12LA, United Kingdom; Department of Environmental Science, Aarhus University, Roskilde DK-4000, Denmark; The Soil Stars, Applied Microbiology International, Cambridge CB12LA, United Kingdom; Department of Microbiology, The Ohio State University, Columbus, OH 43210, United States; EMERGE Biology Integration Institute, The Ohio State University, Columbus, OH 43210, United States; Center of Microbiome Science, The Ohio State University, Columbus, OH 43210, United States; Department of Civil, Environmental and Geodetic Engineering, Ohio State University, Columbus, OH 43210, United States

**Keywords:** microbial inoculants, soil restoration, microbiome engineering, ecological risk assessment, release-based stewardship

## Abstract

Global soil degradation and increasing reliance on chemical inputs threaten agricultural sustainability, driving interest in microbial inoculants as tools for soil restoration. These biological products have the potential to enhance nutrient cycling, improve soil structure, and support plant resilience, but their environmental release raises important safety and stewardship considerations. Here, we propose a risk-proportional framework for the responsible deployment of microbial inoculants grounded in release-based stewardship. The framework integrates genome-resolved strain identification, exclusionary hazard screening, bioassay-based risk triage, ecological testing under realistic conditions, and monitored field deployment. Drawing on evidence from microbial ecology and invasion biology, we highlight how inoculants can alter resident microbial communities, influence ecosystem function, and, in some cases, facilitate gene flow, underscoring the need for risk assessment. We further outline a federated, genome-informed data infrastructure to support traceability, cross-jurisdiction learning, and adaptive management. Together, this approach provides a scalable and scientifically grounded pathway to balance innovation and safety, enabling microbial technologies to contribute to soil restoration and climate-resilient agriculture.

**Sustainability Statement**

This work primarily supports SDG 15: Life on Land by advancing a risk-governed framework for the responsible use of microbial inoculants in soil restoration. The approach is designed to help recover degraded soils, protect ecosystem functions, and support biodiversity by integrating genomic screening, ecological testing, and post-release monitoring. By enabling safer adoption of biological alternatives to intensive chemical inputs, the framework may contribute to more sustainable land stewardship and regenerative agriculture. Additional relevant SDGs: SDG 2 (Zero Hunger) and SDG 13 (Climate Action).

## From containment to release-based stewardship

The degradation of global soil systems has reached a critical point. Conventional management practices, characterized by heavy chemical fertilization and intensive tilling, have disrupted natural microbial processes and degraded soil structure (Albright et al. 2022). Projections suggest that without intervention, global topsoil may be largely lost by 2050 (Kaminsky et al. 2019). This urgency is driving rapid interest in microbial inoculants as a tool that may contribute to soil restoration efforts. Continued reliance on intensive chemical inputs carries well-documented cumulative risks including soil acidification, nutrient runoff and eutrophication, and suppression of beneficial soil microbiota and meiofauna, that are difficult to reverse (Li et al. 2024). Microbial inoculants offer an alternative strategy. By introducing living, soil-associated organisms (typically not genetically modified), microbial inoculants have the potential to restore or reinforce core biological functions such as nutrient cycling, root−microbe interactions, and soil aggregation (Mawarda et al. 2020, Albright et al. 2022). However, the intentional release of living organisms into complex ecosystems raises legitimate safety and stewardship questions. Past biological introductions (intentional or not) have caused substantial ecological harm, e.g. the spread of the chytrid fungus *Batrachochytrium dendrobatidis*, which decimated amphibian populations worldwide, as well as numerous nonmicrobial examples where biocontrol measures have failed (Schulz et al. 2019, Scheele et al. 2019). These precedents underscore that microbial technologies cannot be assumed to be risk-free and require rigorous, context-appropriate oversight. In 1975, the landmark Asilomar Conference established a precedent for managing biological risk (Berg 2008). The 2025 “Spirit of Asilomar” summit extended this to open-environment releases, recognizing that microbial soil amendments (Beattie et al. 2025) require what we term release-based stewardship (Chemla et al. 2025).

Release-based stewardship blends traditional prerelease planning with considerations for post-release impacts and risks, involving strain-resolved genomic screening, ecological testing, confined and traceable field evaluation, and post-release monitoring. Implementing this approach is complicated by a fragmented regulatory landscape in which oversight is split across agricultural, environmental, and public health authorities, resulting in duplicate testing and limited data portability (Kaminsky et al. 2019). We propose a unified framework for rigorous yet scalable stewardship of microbial inoculants for soil restoration, translating ecological and regulatory principles into actionable guidance for developers, regulators, and practitioners.

Microbial inoculants span a wide spectrum of biological complexity. At one end lie well-characterized single-strain bacterial biofertilizers, such as *Rhizobium* and *Bradyrhizobium* spp., with decades of use and well-established safety profiles. At the other end lie defined microbial consortia and emerging microbiome-engineering platforms designed to reprogram soil functional networks. Fungal inoculants, including arbuscular mycorrhizal fungi, have distinct risk profiles: AMF have broad plant host ranges but, as obligate biotrophs, require a living host for active growth and reproduction, which limits their capacity for sustained independent establishment beyond the deployment site. The stewardship framework proposed here applies to all these categories, but the level of scrutiny required at each decision gate scales explicitly with inoculant complexity, ecological novelty, and the availability of prior safety data. A low-complexity, nonrecombinant bacterial strain with no known virulence factors, restricted persistence, and a well-documented environmental track record may proceed through abbreviated Gate 1 screening; a defined or engineered natural consortium designed for large-scale landscape restoration requires full-scale genomic, ecological, and monitoring review.

## Inoculant impacts, persistence, and legacy effects

Microbial inoculants can alter the composition and function of recipient soil communities, sometimes beneficially, often transiently, and in some cases with effects that persist beyond the intended window of application (Mawarda et al. 2020, Li et al. , Zhang et al. 2026, Cornell et al. 2021, Francioli et al. 2025, Liu et al. 2023). In fact, measurable shifts in the composition or function of soil microbial communities can occur even when inoculant persistence is limited. At the same time, many soils exhibit strong biotic resistance, where established microbial communities limit invasion and constrain long-term establishment of introduced strains, particularly in healthy, undisturbed systems, where high microbial diversity hinders potential impacts (van Elsas et ak. 2012), Shokri et al. 2025), Klümper et al. 2024). Recognizing this distinction is central to responsible stewardship because it underscores the need to evaluate both short-term functional outcomes and potential longer-term ecological effects when assessing inoculant performance and risk (Mallon et al. 2018).

### Inoculants reshape microbial community composition and genetic diversity

The deliberate introduction of microbial inoculants frequently alters resident soil microbial communities. In a meta-analysis of 108 studies (Mawarda et al. 2020), 86% showed that inoculants modified the composition of resident microbial communities. Sometimes these shifts appeared to be beneficial. For instance, application of a biofertilizer containing *Bacillus amyloliquefaciens* W19 and *Trichoderma guizhouense* NJAU4742 increased the abundance of taxa with potential antagonistic activity toward plant pathogens (Xiong et al. 2017). Other times, potentially less desirable effects were noted. For instance, the release of *Sinorhizobium meliloti* L33 into the rhizosphere reduced the diversity of beneficial *Pseudomonas* species (Schwieger and Tebbe 2000). Beyond compositional changes, inoculants can influence the genetic structure of resident microbial populations through horizontal gene transfer (HGT). Because HGT is a well-known route for the dissemination of antimicrobial resistance and other high-consequence traits, it is appropriately viewed as a potential risk mechanism and warrants explicit, strain-resolved scrutiny. For example, repeated large-scale inoculation of soybean with commercial *Bradyrhizobium* strains in Brazil resulted in extensive horizontal transfer of symbiotic genes to resident rhizobia (Barcellos et al. 2007, Batista et al. 2007). These observations show that inoculants can act as vectors of functional trait movement, sometimes beneficial (e.g. spreading symbiotic capacity), but potentially harmful if mobile elements carry antimicrobial resistance, toxin, or virulence-associated loci. Accordingly, HGT risk should be treated as feature- and context-dependent: prioritized for strains with mobility-associated machinery or flagged loci during genome screening and verified through targeted field monitoring in premarket trials.

In many cases, shifts in community composition translate into measurable changes in soil functioning. Across multiple studies, inoculation was found to increase activities of phosphatase, sulfatase, chitinase, esterase, urease, and other enzymes thus favorably impacting nutrient cycling, fertilization, decomposition, and biocontrol (Vázquez et al. 2000, Wu et al. 2005). For example, in one study, the introduction of *Paenibacillus mucilaginosus* 3016 was correlated with increased soil phosphatase enzyme activity (Ma et al. 2018), which resulted in increased phosphate mobility and availability for plant growth. In other studies, inoculation was associated with the emergence of disease-suppressive soils (Shen et al. 2014, Xiong et al. 2017), even if the inoculant does not survive or drops below levels of detection (Deng et al. 2021), underscoring the potential for inoculants to steer functional outcomes by driving microbial community reassembly through secondary succession.

### Persistence constraints and legacy effects

While inoculants may initially reach high abundance, they typically decline over time and stabilize at low levels (<1%) within weeks (Čaušević et al. 2022)). This suggests integration into existing communities rather than long-term dominance, although functional effects can still occur at low abundance through metabolic interactions or signaling. These ecological constraints can limit persistence but should not be assumed as safety controls, as they are context-dependent and require empirical validation. Inoculant persistence can be restricted by lack of local adaptation, competition, predation, and niche saturation (Berg 2008, Dong et al. 2024, Marken 2025). Establishment is highly context-dependent, with establishment often more successful in disturbed or degraded soils than in healthy soils, which are more invasion resistant (Hibbing et al. 2010, Klümper et al. 2024). These dynamics should be evaluated within a structured monitoring framework.

Even when inoculant populations decline, their ecological effects can persist. Studies show lasting shifts in community composition despite rapid decreases in inoculant abundance (Kozdrój and van Elsas 2004, Mallon et al. 2018). For example, *Sinorhizobium meliloti* L33 altered rhizosphere communities and dispersed beyond treated plots (Schwieger and Tebbe 2000). These findings highlight that inoculants can reshape microbial communities and spread beyond target sites, with both beneficial and unintended consequences, underscoring the need to assess community-level responses in risk evaluation.

## Prerelease screening and field stewardship

**Figure 1:**
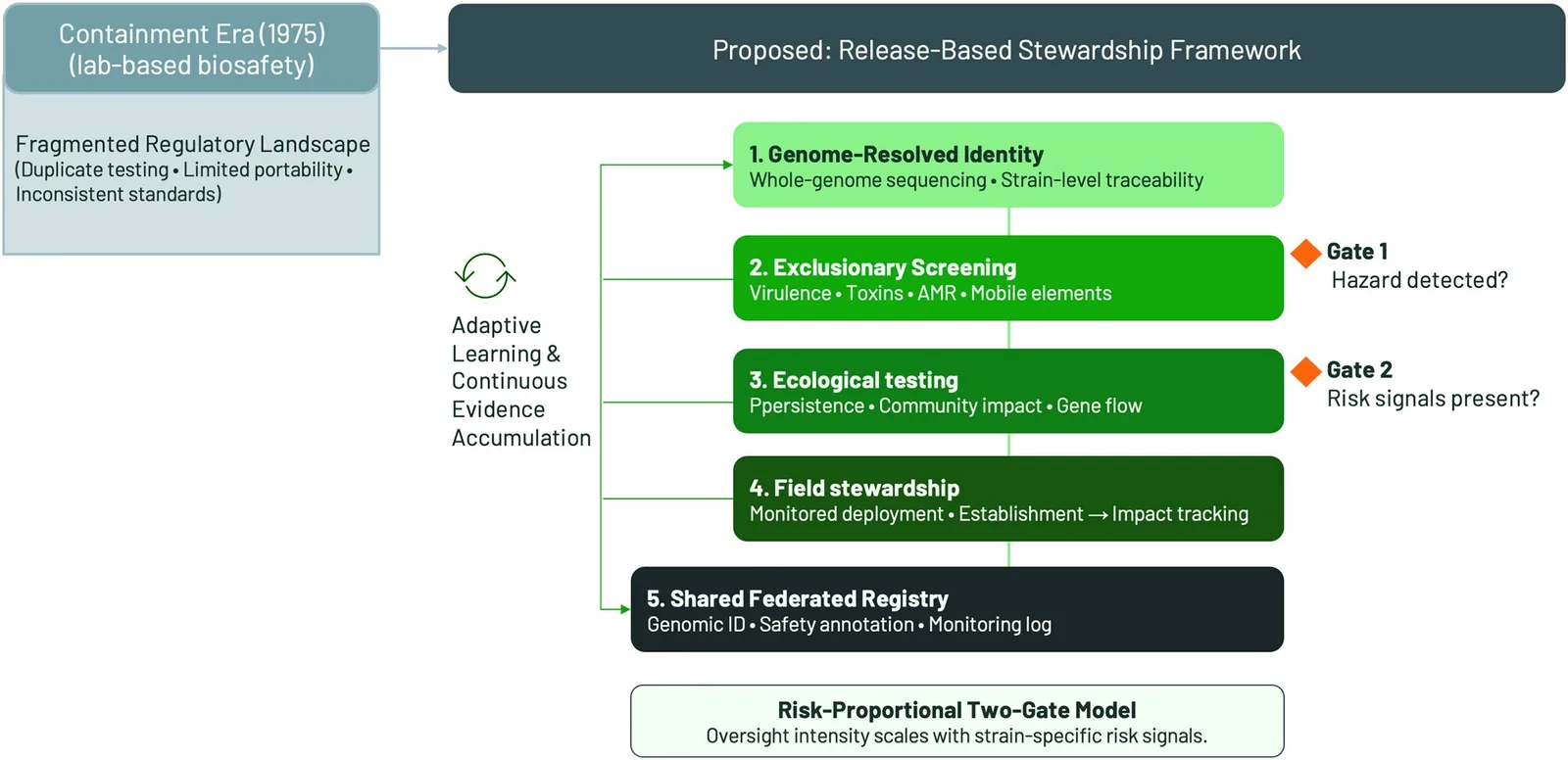
Risk-proportional release-based stewardship framework for microbial inoculants. Candidate strains undergo genome-based identity confirmation and hazard screening, followed by ecological testing and monitored field evaluation. Oversight is scaled to strain-specific risk signals, and a federated registry supports traceability, shared learning, and adaptive stewardship. The most serious failure for the field would be an inoculant linked to disease in crops, livestock, wildlife, or humans. Even rare events would undermine public trust and regulatory confidence, so risk assessment should begin with exclusionary screening that disqualifies strains with high-consequence hazard traits regardless of efficacy. Although consortia can in principle generate emergent behaviors through microbial interactions, hazards such as virulence, toxin production, and clinically relevant antimicrobial resistance are largely strain-encoded and can usually be identified through genome-resolved screening (Steps 1 and 2, Fig. 1). Still, because interactions within consortia may affect persistence and ecological function, the framework includes consortia-level ecological testing and monitored field evaluation to verify that no unintended risks emerge under realistic conditions (Steps 3 and 4, Fig. 1).

**Figure 2:**
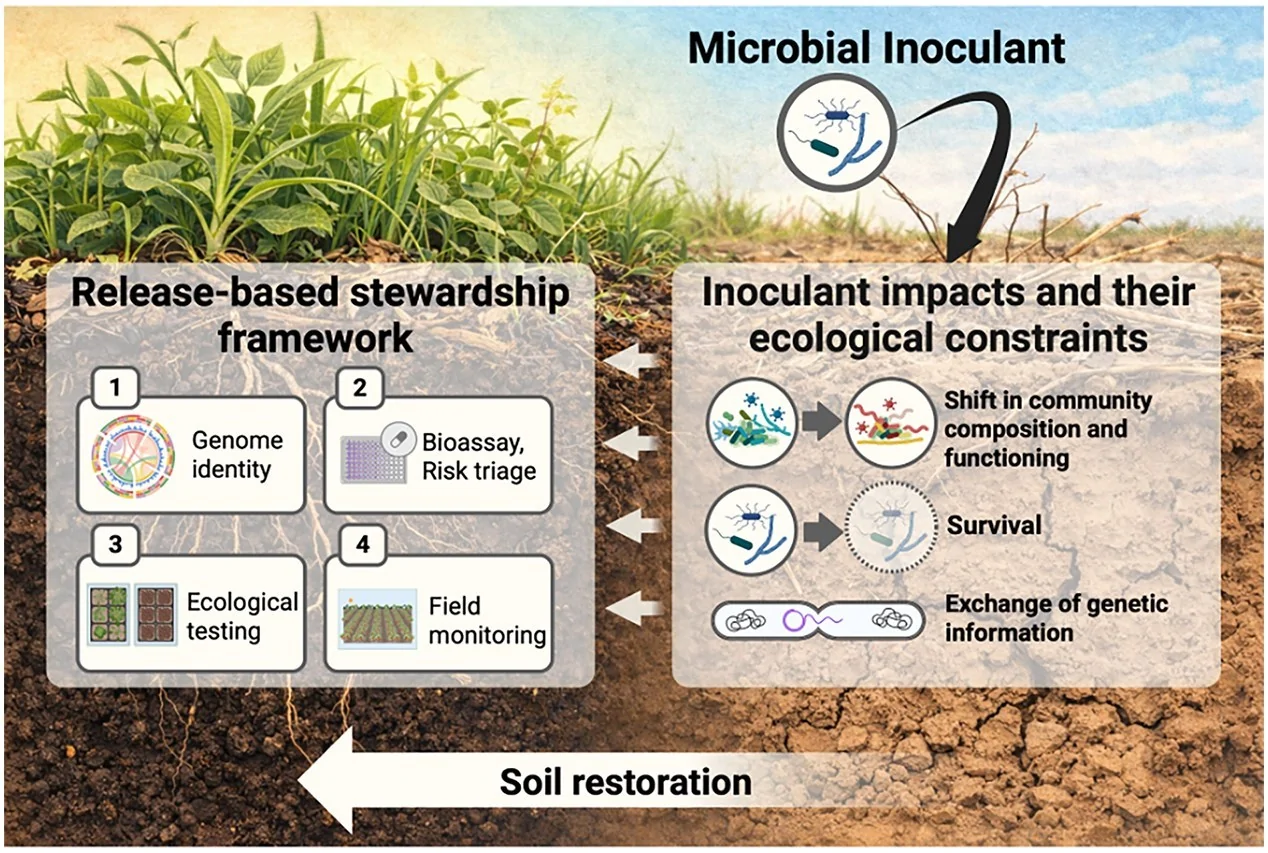
Release-based stewardship framework for microbial inoculants in soil restoration. Steps: (1) genome-resolved strain identification and hazard screening, (2) bioassay-based risk triage, (3) ecological testing under realistic conditions, and (4) monitored field deployment.

We propose a two-phase framework for the responsible deployment of microbial inoculants: prerelease risk screening followed by field stewardship (Fig. 1 and Fig. 2). A prerequisite for implementing this framework is validation that the inoculant contains the declared microbial species and not nondisclosed organisms or additives. Product quality control failures are well-documented [e.g. many commercial AMF inoculants do not contain the species stated by the manufacturer (Boussageon et al. 2025)]. Such failures must be addressed before risk assessment proceeds.

### Steps 1 and 2: Prerelease risk screening: genome identity and hazard/bioassay triage

Step 1 is resolving the identity of the strain and its relationship to high-concern clades. Whole-genome sequencing (WGS) should be the baseline for commercial candidate strains because marker sequences such as 16S rRNA, 18S rRNA, and the internal transcribed spacer (ITS) often lack the resolution to distinguish lineages with different hazard potentials. WGS enables strain-level identification and placement relative to known pathogenic or regulated clades using curated reference databases and genome-to-genome comparisons such as Average Nucleotide Identity (ANI) (Konstantinidis and Tiedje 2005). Lineage-aware tools can further improve classification in high-concern groups (Zhang et al. 2026). However, phylogenetic distance from known pathogens should not be taken as evidence of safety, since distantly related strains may still carry virulence-, toxin-, resistance-, or mobility-associated traits that require further screening.

Step 2 is generating evidence for risk based on WGS data and establishing context to triage downstream testing. A targeted literature and evidence review can inform interpretation of the hazards posed by taxonomic placement in Step 1 (e.g. whether a clade has documented pathogenic members) and help triage the depth of downstream genomic scrutiny. Strains distantly related to known pathogenic clades may still carry virulence-associated, resistance, or mobility-linked features that require flagging and can be evaluated in targeted bioassays. Because virulence and antimicrobial resistance traits can spread through mobile genetic elements, candidate genomes should be screened for mobility-linked hazard potential, not just evidence of past HGT (Partridge et al. 2018). Screening should identify mobile elements such as plasmids, integrative elements, transposons, integrons, and prophages, and determine whether they carry clinically or agriculturally relevant resistance, virulence, or toxin genes. Targeted assays should also assess evidence of active mobility. Candidates carrying mobile elements with high-consequence traits should be excluded or subjected to heightened scrutiny, whereas those lacking such traits may proceed with documentation and monitoring.

Step 2 also involves integrating standardized ecotoxicology and biosafety assays (e.g. impacts on representative soil fauna, beneficial insects, aquatic proxies, nontarget plants, and small mammals) into a composite interpretive framework such as the Environmental and Human Safety Index (EHSI) (Vílchez et al. 2016). Such assays are already widely used in microbial biopesticide and biostimulant evaluation (e.g. OECD/EPA guideline tests). Under a risk-proportional model, only the subset of assays relevant to the candidate strain, use pattern, and exposure pathway would be required. The resulting index would provide an interpretable “go/no-go” gate (Fig. 1) aligned with emerging biostimulant regulatory philosophies while remaining feasible for large-scale screening. For example, a nonsporulating *Pseudomonas fluorescens* PGPR strain with no virulence factors, no plasmids, and a predicted 14−20-month survival window in semi-arid loamy sand might score ∼11/50 on the EHSI, clearing Gate 1 and proceeding to confined field evaluation under standard Tier 1 post-release monitoring conditions.

### Steps 3 and 4: Field stewardship: ecological testing and monitored deployment

Stewardship decisions must integrate ecological benefit alongside risk. Applicants should define, a priori, measurable benefit endpoints, such as improvements in SOC accumulation, nitrogen mineralization, phosphate solubilization, aggregate stability, or other soil restoration traits, or agricultural yield improvement and fertilizer use efficiency. Community-level benefit must also be assessed: native diversity should be maintained or enhanced and keystone taxa not displaced. Benefit endpoints should be evaluated alongside EHSI safety scoring to determine whether expected benefit justifies residual risk.

Even when acute biosafety risks are excluded, microbial inoculants may still cause unintended ecological effects, particularly in multistrain consortia where interactions can alter persistence, functional expression, or impact relative to single strains. In steps 3 and 4 of the framework, we therefore apply an invasion-ecology framework in which risk is evaluated as a progression from introduction to establishment, spread, and impact (Mallon et al. 2015). Here, impact refers specifically to unintended ecological disruption rather than intended agronomic or restoration benefits. Ecological stewardship should therefore assess whether deployment leads to adverse outcomes such as displacement of native beneficial taxa, unexpected persistence, altered nutrient-cycling trajectories, destabilization of disease-suppressive communities, horizontal transfer of high-consequence traits, or off-site movement into nontarget habitats.

## The risk-benefit balance

The analogy to invasion ecology provides a valuable lens for anticipating unintended establishment, competitive displacement, and community restructuring following inoculant release. However, the analogy has important limits in the restoration context. Classical invasion biology is concerned with the unintended arrival of organisms into naïve ecosystems. Intentional microbial inoculants, by contrast, are deployed precisely because the target ecosystem has been ecologically impoverished through anthropogenic disturbance, and the goal is to restore lost functions. This distinction is normatively significant. The relevant question is not whether the inoculant establishes, but whether it does so in a way that is ecologically bounded, functionally coherent, and compatible with the eventual recovery of native microbial communities. The Two-Gate Model therefore does not aim to prevent all establishment but to distinguish between bounded, function-serving establishment and unbounded, ecologically disruptive spread. Where invasion ecology is most directly applicable is in characterizing post-release dynamics and in providing validated metrics, such as propagule pressure, competitive exclusion assays, and dispersal modelling, for the monitoring phase.

## Soil environmental context as a risk modifier

Risk is not a fixed property of the microorganism alone but an emergent property of organism–environment interaction. Soil texture, pH, organic matter, moisture, and resident microbiota all modulate persistence, competitive dynamics, and dispersal. Degraded soils—the primary targets of restoration inoculants—are simultaneously low-colonization-resistant environments (enabling establishment) and ecologically impoverished systems where loss of microbial functional guilds may predispose them to functional takeover by dominant introduced strains. EHSI scoring incorporates a soil vulnerability assessment calibrated to the target deployment pedology; applicants must provide basic soil characterization data (texture class, pH, TOC, bulk density) as part of the precommercial dossier (Shokri et al. 2025).

## Environmental monitoring

**Figure 3:**
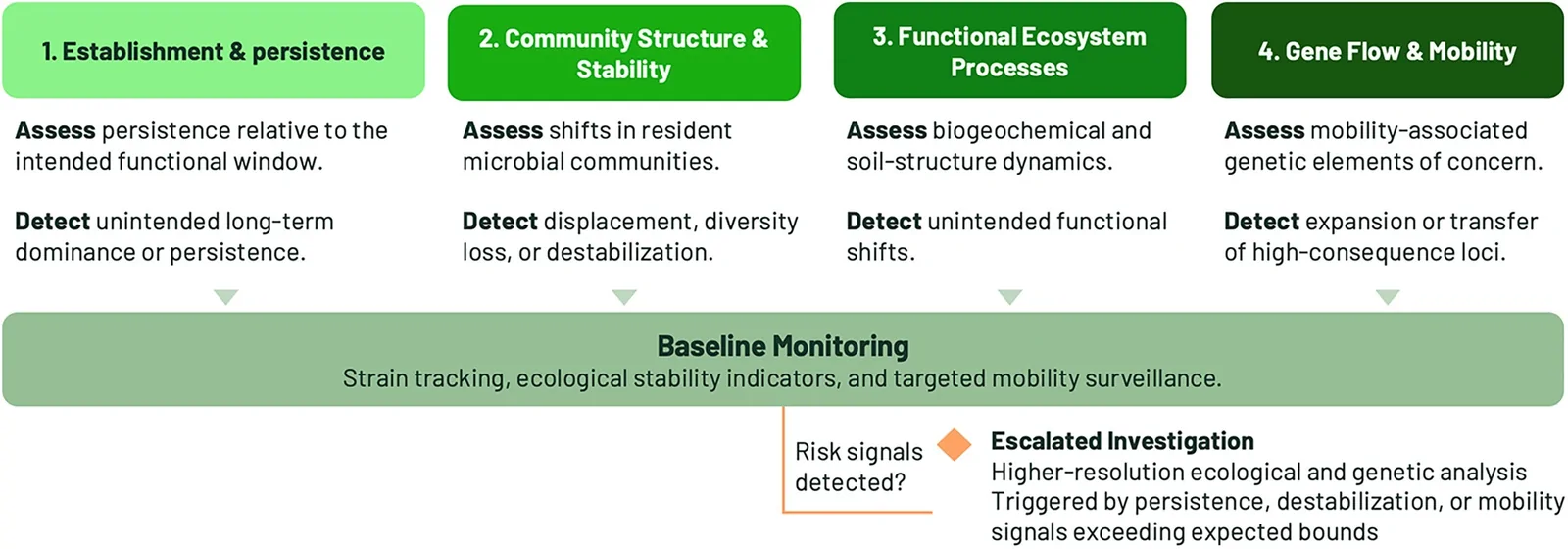
Conceptual framework for tiered environmental monitoring of microbial inoculants after field release.

Premarket field trials should be conducted under realistic conditions while remaining geographically bounded to allow traceability and monitoring. Their deployment should be controlled within defined spatial parameters with strain-resolved traceability. These trials should evaluate both intended agronomic performance and potential ecological risk indicators. Monitoring should be structured around four core domains as follows (Fig. 3):

(I) Establishment and Persistence.

Strain-resolved detection (e.g. qPCR or genome-resolved omics) should confirm establishment, track abundance over time, and identify unexpected long-term dominance (McMullen and Lennon 2023).

(II) Community Structure and Stability.

Microbiome profiling should assess shifts in resident community diversity, stability, and composition, particularly under repeated applications or in sensitive systems.

(III) Functional Ecosystem Processes.

Indicators including soil respiration, nitrogen cycling, and pathogen suppression should distinguish beneficial functional shifts from unintended changes.

(IV) Gene Flow and Mobility.

Gene-flow monitoring should be applied to strains with mobility-associated elements or flagged resistance or virulence genes; field monitoring should verify that these do not expand or transfer under agronomic conditions.

Recognizing practical constraints, we propose a tiered, risk-proportional approach where baseline monitoring is broadly applied and additional measures are triggered by genomic features, deployment context, or claim requirements. At minimum, trials should include strain-resolved tracking and a targeted mobility watchlist. Monitoring can escalate where elevated risk signals are detected. All trials should include appropriate controls to distinguish inoculant effects from background variability.

### Post-market monitoring and adaptive stewardship

Because soils are spatially and temporally dynamic, stewardship should extend beyond initial field validation. Post-market monitoring should verify that abundance trajectories align with the intended use profile and that functional outcomes remain within expected bounds. Persistence is not inherently undesirable: for some applications (e.g. symbiotic functions) establishment may be necessary for efficacy; monitoring should evaluate persistence relative to product intent rather than a single universal expectation.

Complete spatial containment of microorganisms in open agricultural systems cannot be assumed; microbial movement via wind, water, and biotic vectors is well established. Post-market monitoring aims to verify that any dispersal remains ecologically benign and consistent with upstream risk assessments. Harmonized monitoring frameworks and standardized global ecological risk metrics remain an important development priority.

### Authorization, adaptive governance, and implementation

Where high efficacy accompanies low but nonzero ecological risk, a conditional authorization pathway allows limited commercial deployment subject to: (i) mandatory post-market ecological monitoring at sentinel sites; (ii) dose minimization to achieve efficacy at the lowest effective inoculum density; and (iii) predefined thresholds triggering reassessment, suspension, or withdrawal. Efficacy and safety data must be submitted together; regulators should treat both as complementary axes of evidence.

Post-release monitoring feeds into a three-tier adaptive governance system: Tier 1 (Watch), unexpected spread or significant diversity reduction triggers increased monitoring; Tier 2 (Review), persistence of Tier 1 conditions or HGT of resistance determinants triggers formal regulatory review within 90 days; Tier 3 (Withdrawal), persistent community restructuring or ecosystem function impairment triggers mandatory suspension. Thresholds are defined prospectively and published in the authorization record.

Full strain-resolved genomic characterization is required once per novel strain as a developer investment, not a recurring end-user cost. Batch QC for established strains requires only amplicon-based identity confirmation and viability testing. Strains approved under an equivalent regulatory framework elsewhere may access expedited review through data portability. Post-release community monitoring is appropriately conducted using 16S rRNA amplicon sequencing rather than continuous full shotgun metagenomics (Díaz-Rodríguez et al. 2025).

## A federated registry for inoculant stewardship

Effective stewardship requires knowing which strain is being released and what safety evidence exists. Current oversight is fragmented, leaving data siloed and duplicating studies. We propose a federated global registry linking strain-resolved genomic identifiers with risk screening, ecological testing, field monitoring, and regulatory outcomes (Table 1). This registry would make safety data discoverable and portable while supporting cross-jurisdiction learning and adaptive stewardship.

**Table 1:** Proposed federated registry architecture for inoculant stewardship

Layer	Purpose	Core components	Why it matters
**1. Genomic identifier layer (anchor)**	Establish strain-resolved identity	• Whole-genome sequencing (WGS) • Reference genome/assembly version + QC metrics • ANI thresholds • Versioned taxonomy alignment • Deposit/location of sequences	• Prevents misidentification • Enables exclusionary hazard screening • Ensures traceability across jurisdictions • Prevents “same strain ID, different genome” problems
**2. Stewardship & provenance metadata**	Contextualize strain use	• Strain origin • Intended-use environment • Intended functional duration • Formulation • Deployment geography • Application method + dose • Crop + soil type + management	• Risk is context-dependent—ecological behavior cannot be interpreted without use metadata
**3. Risk-screening repository**	Archive exclusionary hazard screening	• Virulence-factor screening • ARG database outputs • Toxin genes • AMR loci • Mobility-linked elements • Bioassay outputs	• Provides transparent “go/no-go” documentation • Reduces redundant testing across regions
**4. Ecological testing & performance data**	Evaluate establishment and impact	• Persistence/decline kinetics • Baseline and controls definition • Sampling design metadata • Spread assessments • Community composition shifts • Nutrient-cycling indicators • Nontarget effects • Functional outcomes	• Distinguishes intended benefits from unintended ecological disruption • Makes results comparable—avoids “apples vs oranges
**5. Regulatory & monitoring log**	Enable reciprocity and adaptive governance	• Prior regulatory decisions • Approval conditions • Post-market monitoring data • Time-stamped updates • Adverse event reporting/incident log (even if rare) • Versioning of regulatory status	• Allows safety profile to travel with strain • Supports cross-border harmonization and long-term oversight • Provides adaptive stewardship system

To protect intellectual property, we propose a tiered disclosure architecture: Tier 1 (open access) covers phenotypic safety endpoints, risk classifications, monitoring summaries, and authorization status; Tier 2 (regulatory access only) covers proprietary genomic identifiers and formulation data, accessible only to competent authorities under confidentiality agreements. Registry architecture follows a federated node model with secretariat functions embedded in an existing international body. Data portability must be bounded: laboratory characterization data are portable within defined pedoclimatic zones, but field-level ecological and efficacy data must be validated locally before informing regulatory decisions in distinct biogeographic contexts.

Soil restoration cannot wait. Microbial inoculants offer real potential to improve crop resilience and accelerate ecosystem recovery, but that potential will only be realized through coherent, strain-resolved stewardship. We call for a shift from fragmented regulation toward a release-based, risk-proportional framework that combines genome-resolved identity, exclusionary safety screening, contextual ecological evaluation, and monitored field deployment, with outcomes captured in shared infrastructure so knowledge accumulates across projects and jurisdictions. Microbial inoculants should be governed neither as inherently safe nor inherently suspect, but as context-dependent restoration tools whose responsible use depends on strain identity, ecological setting, and long-term stewardship.

## Data Availability

No other data source.
